# Isolation Hospitals

**Published:** 1902-02-15

**Authors:** 


### ISOLATION HOSPITALS.

Messrs. Humphreys, the well-known builders of iron hospitals, have prepared a catalogue of isolation hospitals, which is of particular value at the present time, when local authorities are likely to be called upon to act with promptitude and despatch to prevent the spread of small-pox. An instance of this has just occurred at Uxbridge, where the medical officer reports that the isolation hospital has saved the town, for all the cases occurred within a period of six days, and no permanent building could have been erected in the time. The hospital in question was one of Messrs. Humphreys', who have the means for not only putting up the shell, but furnishing throughout, down to the details of bed-linen and crockery, so that within a few days of the first report of the outbreak the hospital may be occupied and in full swing, the building being despatched from the workshops on the same day on which the order is received.

Further qualities and advantages claimed for this form of building are durability, stability, and simplicity, with facility of erection and removal, cleansing and disinfecting. The buildings are, moreover, "fitted for all climates." The foundation required is of about four-course brickwork, with air-bricks and damp-proof course, and ground-plans are supplied for the guidance of the local bricklayer, who can thus prepare the foundation and effect a saving of time. The corrugated iron used is of the best No, 24 B.W. gauge, well galvanised, without flaws or cracks, thickly coated with pure Silesian spelter. The ridge of the roof has a capping so fixed as to allow ventilation throughout the length of the building. The wall-framings are of timber, morticed and tenoned, and can be easily taken down without injury to the building. Matchboarding is used for lining the walls, and there is, in addition, an interlining of felt; the vitiated air makes its escape through holes in the roof-ridge, 6 inches apart. The floors are of planed boards, grooved and tongued. There are roof-dormers for ventilation, and the windows have side-wings to prevent a down draught. An air-space of several inches is allowed for between the boarding, purlins and iron, with a view to keeping the ward at an even temperature. Additions can easily be made to a building by the patented system of construction in use with this firm, and the proportionate cost is not great. The ease with which these hospitals can be taken to pieces has this great advantage, that if the site fixed upon is found on experience to be unsuitable, or if it ceases, on account of other buildings, to be sufficiently isolated for the required purpose, it can be removed to a more convenient site without injury to the structure.

These hospitals are made in ten different sizes—from the mere ward, as an addition to an already existing hospital, to the complete block, the latter having doctors', matrons', and nurses' rooms, kitchen and offices, and a corridor connecting the wards. When the administrative block can be made separate from the main building Messrs. Humphreys recommend that it should be so, and that the approach should be by a covered corridor open at the sides. In their plans the mortuary, laundry, disinfecting chamber, and an isolation ward for doubtful cases are entirely apart from the wards and built in the form of outhouses, though on precisely the same principles as the main block. The floors of the outbuildings should be of concrete.

With regard to the cost of their hospitals, Messrs. Humphreys state that in districts where it is not desired to levy a special rate, they are willing to accept payment by instalments spread over an agreed period. They add that frequently the interest paid on borrowed money for a brick building would have defrayed the cost of an iron one. The cost for maintenance, also, is very much lower in the case of the temporary hospitals.

As to warming the wards, the method to be employed is usually left to the choice of the surveyor, who is also responsible for the drainage, only gutters and down pipes to the ground level being included in the estimate. The necessary warmth may be obtained by hot air, steam, or hot water, high or low pressure; but the open stove is the form recommended. This costs from £5 to £10, according to size, and should be placed in the middle of the ward, on a stone slab, with a flue carried up through the roof; and an iron flue protector should be added.

